# Cognitive decline among older adults with heart diseases before and during the COVID-19 pandemic: A longitudinal cohort study

**DOI:** 10.3389/fcvm.2022.1077800

**Published:** 2023-01-26

**Authors:** Rong Hua, Chenglong Li, Darui Gao, Fanfan Zheng, Wuxiang Xie

**Affiliations:** ^1^Peking University Clinical Research Institute, Peking University First Hospital, Beijing, China; ^2^Peking University Clinical Research Institute Heart and Vascular Health Research Center at Peking University Shougang Hospital, Beijing, China; ^3^School of Nursing, Peking Union Medical College, Chinese Academy of Medical Sciences, Beijing, China

**Keywords:** heart diseases, cognitive decline, dementia, COVID-19 pandemic, older adult

## Abstract

**Background:**

Little is known about the impact induced by the COVID-19 pandemic on the cognitive function of older adults with heart diseases. This study aimed to examine whether older adults with heart diseases suffered larger cognitive deterioration during the COVID-19 pandemic.

**Methods:**

This study leveraged longitudinal data from the Health and Retirement Study (HRS), a nationally representative U.S. aging cohort with objective cognitive assessments measured before and during the pandemic. The interval from HRS waves 13 to 14 (April 2016 to June 2019) was defined as the pre-pandemic period to control the pre-existed cognitive difference between participants with and without heart diseases, and the interval from waves 14 to 15 (June 2019 to June 2021) was defined as the pandemic period. The HRS wave 14 survey was considered the baseline. The heart disease status was defined by a self-reported diagnosis. Linear mixed models were performed to evaluate and compare the cognitive differences during different periods.

**Results:**

A total of 9,304 participants (women: 5,655, 60.8%; mean age: 65.8 ± 10.8 years) were included, and 2,119 (22.8%) had heart diseases. During the pre-pandemic period, there was no significant difference (−0.03, 95% CI: −0.22 to 0.15, *P* = 0.716) in the changes in global cognitive scores between participants with and without heart disease. During the pandemic period, a larger decreased change in the global cognitive score was observed in the heart disease group compared with the non-heart disease group (−0.37, 95% CI: −0.55 to −0.19, *P* < 0.001). An enlarged difference in global cognitive score was observed during the pandemic period (−0.33, 95% CI: −0.65 to −0.02, *P* = 0.036).

**Conclusion:**

The findings demonstrated that the population with heart diseases suffered more cognitive decline related to the pandemic, underscoring the necessity to provide immediate cognitive monitoring and interventions for the population with heart diseases.

## 1. Introduction

Since 11 March 2020, the World Health Organization has designated the coronavirus disease 2019 (COVID-19) as a global epidemic (1). This pandemic has exerted an unprecedented impact on the multi-dimension of people’s lives. Notably, it has intrigued health concerns on non-communicable diseases due to the constraints on healthcare resources and changes in public mental wellbeing and behaviors (2, 3). It is of vital clinical and public health importance to understand the consequence of the pandemic on non-communicable diseases and to better adapt responses to this persistent pandemic crisis.

Heart diseases are the leading cause of morbidity and mortality in older adults (4). During the pandemic, most outpatient visits, elective procedures, cardiac rehabilitation, and telemedicine programs have been canceled or postponed to prioritize the care of patients with COVID-19 (5, 6). The reduced access to healthcare has affected the vulnerable population with heart disease. Moreover, the enforced social isolation during the pandemic has caused a spectrum of mental disorders and unhealthy lifestyles, which are recognized cardiovascular risk factors and contribute to poorer prognosis in the population with heart diseases (7–10). The European Society of Cardiology has issued guidance for the management of cardiovascular diseases during the COVID-19 pandemic to mitigate the deleterious impact of the pandemic (11). However, it is worthwhile that the adverse outcomes of the pandemic on the population with heart diseases might not be limited to cardiac manifestation. Even before the pandemic, accumulated evidence has proven that older adults with heart diseases exhibit elevated risks of cognitive decline and dementia, potentially owing to multiple mechanisms, including atherosclerotic processes, vascular oxidative stress, and inflammation response (12, 13). The latest American Heart Association (AHA) Heart Disease and Stroke Statistics demonstrated that promoting cardiovascular health would help retain cognitive function and achieve healthy aging (14). The exacerbation of cardiovascular health during the pandemic could further exaggerate cognitive decline among older adults with heart diseases. The existing evidence has indicated a significant decline in cognitive function during the pandemic among older adults (15–17). Still, it is important to further identify the most vulnerable population toward the pandemic-induced cognitive decline for service providers and policymakers.

We, therefore, aimed to examine whether older adults with heart diseases suffered larger cognitive deterioration during the COVID-19 pandemic. The present study was designed in the framework of a well-established U.S. aging cohort. We took advantage of the available objective cognitive assessments measured before and during the pandemic, to account for the existing difference in cognitive function between people with and without heart diseases preceding the pandemic, and thus accurately detecting the impact directly related to the pandemic. We hypothesized that the pandemic would induce an enlarged gap in cognitive function between people with and without heart disease.

## 2. Materials and methods

### 2.1. Study population

The Health and Retirement Study (HRS) is a nationally representative longitudinal cohort study of U.S. community dwellers aged 50 years and older, which has been conducted biennially since 1992. Detailed conception and methods of this study have been well documented elsewhere (18). The HRS was approved by the Institutional Reviewing Board at the University of Michigan and the National Institute on Aging (HUM00061128), and all participants have provided written informed consent.

**FIGURE 1:**
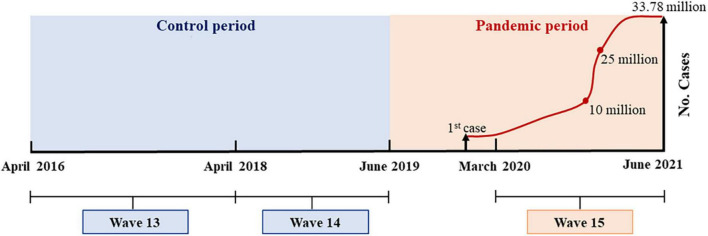
Timeline of Health and Retirement Study (HRS) surveys and cumulative-confirmed coronavirus disease 2019 (COVID-19) cases in the United States.

The timeline of the present study is exhibited in Figure 1. The interval between HRS wave 13 (April 2016 to April 2018) and wave 14 (April 2018 to June 2019) was considered the control period. The interval between wave 14 and wave 15 (March 2020 to June 2021) was considered the pandemic period. The first confirmed COVID-19 case in the United States was reported on 20 January 2020 (19), and the number of cumulative-confirmed cases during HRS wave 15 from 1 March 2020 to 30 June 2021 elevated from 32 to 33.78 million.

HRS wave 14 was considered the baseline. As shown in Supplementary Figure 1, among a total of 17,146 participants who attended the wave 14 survey, we excluded 7,295 participants without complete data on cognitive assessment at any one wave from wave 13 to 15 surveys and 547 participants with existing dementia before the pandemic. Finally, 9,304 participants were included in the present study. Supplementary Table 1 shows the differences in characteristics between included and excluded participants. The excluded participants were significantly older and less healthy.

### 2.2. Heart diseases ascertainment

We identified the heart disease status by the following question in HRS wave 14: “Has a doctor told you that you have had a heart attack, coronary heart disease, angina, congestive heart failure, or other heart problems?” Participants who reported “Yes” were defined as having heart diseases; otherwise, they were regarded as without heart diseases.

### 2.3. Cognitive assessments

The HRS evaluated cognitive function *via* an adapted version of the Telephone Interview for Cognitive Status (20), which is a sensitive screening tool fit for large-scale population-based surveys. The validity and consistency of the HRS cognitive assessments have been well documented (21, 22).

The HRS assessed memory (the immediate and delayed word recall test ranged from 0 to 20 points) and executive function (one was the serial 7’s subtraction test, which ranged from 0 to 5 points to evaluate working memory, and another was the counting backward test, which ranged from 0 to 2 points to evaluate processing speed and attention) on all respondents. The global cognitive score was the summary of two component scores (ranging from 0 to 27 points), and the higher score manifested better cognitive performance. As shown in previous studies, participants with a global cognitive score of fewer than 7 points were regarded as having dementia (21, 23). The definition of dementia in the present study was a self-reported diagnosis of dementia with a global cognitive score of fewer than 7 points.

### 2.4. Covariates

Potential confounders commonly associated with heart diseases and cognitive decline were selected as *a priori* based on the previous literature (12, 24, 25). These included age at baseline (years), sex, race, educational level, cohabitation status, current lifestyle including smoking, drinking, and physical activity, depressive symptoms, and status of chronic diseases including hypertension, diabetes, stroke, cancer, and chronic lung diseases. Race was divided as white ethnicity or not. A high educational level referred to those who received an education of 12 years or above. The cohabitation status was categorized as living alone at present or not. Participants were categorized into current drinkers (no less than once a week) and non-drinkers (including ex-drinkers), as well as current smokers and non-smokers (including ex-smokers). Physical activity was defined as engaging in weekly moderate or vigorous physical activities at least once. Depressive symptoms were evaluated by using 8-item Center for Epidemiologic Studies Depression Scale (CES-D, the total score ranged from 0 to 8 points), consistent with prior studies (26, 27), and participants who scored 4 or above were regarded as having depressive symptoms. Hypertension was defined as systolic blood pressure of ≥140 mm Hg or diastolic blood pressure of ≥90 mmHg, or self-reported diagnosis of hypertension or use of anti-hypertension drugs. Diabetes was defined as an HbA_1*c*_ of ≥6.5% or self-reported diagnosis of diabetes or the use of anti-diabetic therapy. Other identifications of chronic diseases were based on self-reported diagnoses.

### 2.5. Statistical analysis

The results were presented as the percentage for categorical variables, as well as means ± standard deviations (SD) for continuous variables. Baseline characteristic differences between different heart diseases status were compared by the *t*-test or chi-square test for continuous and categorical variables, respectively.

Linear mixed models were employed to compare the differences in the changes of global cognitive scores by heart disease status during the pre-pandemic period and pandemic period. We adjusted all the covariates mentioned earlier in the linear mixed model. Heart disease status and time were included as classified variables in the model. Time = 0, 2, and 4 were referred to as waves 13, 14, and 15 of the HRS, respectively. At first, least square means (LSMs) and 95% confidence intervals (CIs) after multivariable adjustment of global cognitive scores by heart disease status and time were derived from models. Then, LSM differences in global cognitive scores between heart diseases status at each wave were calculated, and thus, the differences between heart diseases status in the changes of global cognitive scores during the pre-pandemic period and the pandemic period could be estimated, respectively. Finally, we considered the pre-pandemic period as the reference and determined whether the difference between heart disease status in the changes in global cognitive scores during the COVID-19 pandemic period was larger.

In addition, we also repeated the analysis on every single cognitive domain. In sensitivity analysis, we explored potential modified effects of covariates and COVID-19 infection which was defined as the participant self, or his relatives or friends had an infection of COVID-19, on the differences in global cognitive scores between people with and without heart diseases during the pandemic period compared with those during the pre-pandemic period. *Z*-test was applied to examine interaction effects between different subgroups (28).

All analyses were conducted by SAS 9.4 software (SAS Institute Inc., Cary, NC, USA), and a two-sided α value of 0.05 was considered as statistical significance.

## 3. Results

### 3.1. Baseline characteristics

A total of 9,304 participants (women: 5,655, 60.8%; mean age: 65.8 ± 10.8 years) who attended the HRS wave 13–15 surveys were included in the present analysis. All of them have completed cognitive assessments in each of the three waves. There were 2,119 participants with heart disease (22.8%) and 7,185 participants without heart disease (77.2%). The distribution of baseline characteristics by heart disease status is shown in Table 1. Overall, participants with heart diseases were older and had a larger proportion of white ethnicity, with a lower percentage of women, drinking, smoking, and physical activity, while a higher percentage of those having depressive symptoms and chronic diseases exhibited lower global cognitive scores and memory scores.

**TABLE 1:** Baseline characteristics of included participants, by heart diseases status.

Characteristics	Heart disease group (*n* = 2119)	Non-heart disease group (*n* = 7185)	*P* *
Age (years)	70.3 ± 10.8	64.4 ± 10.4	<0.001
Female (%)	1172 (55.3)	4483 (62.4)	<0.001
White (%)	1453 (68.6)	4372 (60.8)	<0.001
High educational level (%)	1706 (80.5)	5800 (80.7)	0.826
Living alone (%)	880 (41.5)	2641 (36.8)	<0.001
Current smoking (%)	249 (11.8)	1010 (14.1)	0.006
Current drinking (%)	696 (32.8)	2916 (40.6)	<0.001
Physical active (%)	1329 (62.7)	5233 (72.8)	<0.001
Depressive symptoms (%)	393 (18.5)	904 (12.6)	
**Chronic diseases status**			
Hypertension (%)	1718 (81.1)	4550 (63.3)	<0.001
Diabetes (%)	881 (41.6)	2062 (28.7)	<0.001
Stroke (%)	346 (16.3)	355 (4.9)	<0.001
Cancer (%)	408 (19.3)	884 (12.3)	<0.001
Chronic lung diseases (%)	405 (19.1)	566 (7.9)	<0.001
**Cognitive scores**			
Global cognitive score	15.3 ± 3.8	16.0 ± 4.0	<0.001
Memory score	9.9 ± 3.1	10.6 ± 3.2	<0.001
Executive function score	5.4 ± 1.7	5.4 ± 1.7	0.638

Data are presented as mean ± SD or n (%).

*The differences between heart disease participants and non-heart disease participants were tested using the *t*-test or chi-square test.

### 3.2. Differences in cognitive changes before and during the pandemic

As shown in Table 2, after adjusting for multiple covariates, in the heart disease group, the LSM of global cognitive scores in wave 13, wave 14, and wave 15 was 15.70 (95% CI: 15.54 to 15.85), 15.91 (95% CI: 15.76 to 16.07), and 15.32 (95% CI: 15.14 to 15.49), respectively. In the non-heart disease group, the LSM in each wave was 15.60 (95% CI: 15.51 to 15.68), 15.85 (95% CI: 15.77 to 15.93), and 15.62 (95% CI: 15.53 to 15.72), respectively. There were no significant differences in global cognitive scores between people with and without heart diseases before the pandemic (wave 13 and wave 14, respectively), while the global cognitive score in the heart disease group was significantly lower than that in the non-heart disease group at wave 15. During the pre-pandemic period, significantly increased changes in global cognitive scores were observed both in the heart disease group and non-heart disease group, respectively. However, no significant difference in the changes in global cognitive scores between people with and without heart diseases was detected (LSM difference: −0.03, 95% CI: −0.22 to 0.15, *P* = 0.716).

**TABLE 2:** Differences in the changes of global cognitive scores before and during the pandemic period, by heart disease status.

Global cognitive scores, LSM (95% CI)*				
	**Heart diseases group (*n* = 2119)**	**Non-heart diseases group (*n* = 7185)**	LSM differences between groups*	*P* for differences between groups*
**Before pandemic**				
Wave 13 (2016)	15.70 (15.54, 15.85)	15.60 (15.51, 15.68)	0.10 (−0.09, 0.28)	0.301
Wave 14 (2018)	15.91 (15.76, 16.07)	15.85 (15.77, 15.93)	0.06 (−0.12, 0.24)	0.491
LSM differences between waves*	0.22 (0.06, 0.38)	0.25 (0.16, 0.34)	−0.03 (−0.22, 0.15)	0.716
*P* for differences between waves*	0.008	<0.001	0.716	/
**During pandemic**				
Wave 14 (2018)	15.91 (15.76, 16.07)	15.85 (15.77, 15.93)	0.06 (−0.12, 0.24)	0.491
Wave 15 (2020)	15.32 (15.14, 15.49)	15.62 (15.53, 15.72)	−0.31 (−0.51, −0.11)	0.003
LSM differences between waves*	−0.60 (−0.76, −0.44)	−0.23 (−0.31, −0.14)	−0.37 (−0.55, −0.19)	<0.001
*P* for differences between waves*	<0.001	<0.001	<0.001	/
**During pandemic vs. Before pandemic**				
Differences in LSM differences between two periods*	−0.81 (−1.09, −0.54)	−0.48 (−0.63, −0.33)	−0.33 (−0.65, −0.02)	0.036
*P* for differences in LSM differences between two periods*	<0.001	<0.001	0.036	/

*Differences were calculated by linear mixed model, after adjusting for age, sex, race, education, cohabitation status, current smoking, current drinking, physical active, depressive symptoms, status of hypertension, diabetes, stroke, cancer, and chronic lung diseases.

During the pandemic period, significant decreases in global cognitive scores from wave 14 to wave 15 were observed in both the heart disease group and the non-heart disease group, respectively. A larger decreased change in global cognitive score was observed in the heart disease group compared with the non-heart disease group (−0.37, 95% CI: −0.55 to −0.19, *P* < 0.001). Compared with the change in the global cognitive score during the pre-pandemic period, people with different heart diseases status exhibited disproportionate cognitive decline during the pandemic: −0.81 (95% CI: −1.09 to −0.54, *P* < 0.001) in the heart disease group and −0.48 (95% CI: −0.63 to −0.33, *P* < 0.001) in the non-heart disease group, respectively. Furthermore, using the cognitive difference between people with and without heart diseases during the pre-pandemic period as the reference, we found that the extent of global cognitive difference among groups was significantly larger during the pandemic period (−0.33, 95% CI: −0.65 to −0.02, *P* = 0.036).

In addition, generally consistent results were yielded in specific cognitive domains. As shown in Tables 3, 4, in the heart disease group, the LSM of memory scores in each wave was 10.15 (95% CI: 10.02 to 10.28), 10.45 (95% CI: 10.32 to 10.57), and 9.97 (95% CI: 9.82 to 10.11), and the LSM of executive function scores was 5.55 (95% CI: 5.48 to 5.62), 5.47 (95% CI: 5.40 to 5.54), and 5.36 (95% CI: 5.28 to 5.43), respectively. In the non-heart disease group, the LSM of memory scores in each wave was 10.14 (95% CI: 10.07 to 10.21), 10.46 (95% CI: 10.39 to 10.52), and 10.26 (95% CI: 10.18 to 10.36), and the LSM of executive function scores was 5.46 (95% CI: 5.42 to 5.49), 5.39 (95% CI: 5.36 to 5.43), and 5.36 (95% CI: 5.32 to 5.40), respectively. There were no significant differences in the changes in memory scores or executive function scores between people with and without heart diseases during the pre-pandemic period. Significantly larger decreased changes were observed in the heart disease group compared with the non-heart disease group in memory scores (−0.28, 95% CI: −0.44 to −0.12, *P* < 0.001), as well as in executive function scores during the pandemic period (−0.09, 95% CI: −0.16 to −0.01, *P* = 0.020). After accounting for the cognitive difference during the pre-pandemic period, people with heart diseases experienced −0.26 (95% CI: −0.54 to 0.02, *P* = 0.068) points of decline in memory scores, as well as −0.07 (95% CI: −0.20 to 0.05, *P* = 0.254) points of decline in executive function scores during the pandemic period.

**TABLE 3:** Differences in the changes of memory scores before and during the pandemic period, by heart disease status.

Memory scores, LSM (95% CI)*				
	**Heart diseases group (*n* = 2119)**	**Non-heart diseases group (*n* = 7185)**	LSM differences between groups*	*P* for differences between groups*
**Before pandemic**				
Wave 13 (2016)	10.15 (10.02, 10.28)	10.14 (10.07, 10.21)	0.01 (−0.14, 0.16)	0.901
Wave 14 (2018)	10.45 (10.32, 10.57)	10.46 (10.39, 10.52)	−0.01 (−0.16, 0.13)	0.881
LSM differences between waves*	0.30 (0.15, 0.44)	0.32 (0.24, 0.39)	−0.02 (−0.18, 0.14)	0.805
*P* for differences between waves*	<0.001	<0.001	0.805	/
**During pandemic**				
Wave 14 (2018)	10.45 (10.32, 10.57)	10.46 (10.39, 10.52)	−0.01 (−0.16, 0.13)	0.881
Wave 15 (2020)	9.97 (9.82, 10.11)	10.26 (10.18, 10.36)	−0.29 (−0.46, −0.13)	<0.001
LSM differences between waves*	−0.48 (−0.62, −0.34)	−0.20 (−0.27, −0.12)	−0.28 (−0.44, −0.12)	0.001
*P* for differences between waves*	<0.001	<0.001	0.001	/
**During pandemic vs. Before pandemic**				
Differences in LSM differences between two periods*	−0.77 (−1.02, −0.53)	−0.51 (−0.65, −0.38)	−0.26 (−0.54, 0.02)	0.068
*P* for differences in LSM differences between two periods*	<0.001	<0.001	0.068	/

*Differences were calculated by the linear mixed model, after adjusting for age, sex, race, education, cohabitation status, current smoking, current drinking, physical active, depressive symptoms, status of hypertension, diabetes, stroke, cancer, and chronic lung diseases.

**TABLE 4:** Differences in the changes of executive function scores before and during the pandemic period, by heart disease status.

Executive function scores, LSM (95% CI)*				
	**Heart diseases group (*n* = 2119)**	**Non-heart diseases group (*n* = 7185)**	LSM differences between groups*	*P* for differences between groups*
**Before pandemic**				
Wave 13 (2016)	5.55 (5.48, 5.62)	5.46 (5.42, 5.49)	0.09 (0.01, 0.17)	0.023
Wave 14 (2018)	5.47 (5.40, 5.54)	5.39 (5.36, 5.43)	0.08 (0.00, 0.16)	0.050
LSM differences between waves*	−0.08 (−0.14, −0.01)	−0.07 (−0.10, −0.03)	−0.01 (−0.09, 0.06)	0.720
*P* for differences between waves*	0.017	<0.001	0.720	/
**During pandemic**				
Wave 14 (2018)	5.47 (5.40, 5.54)	5.39 (5.36, 5.43)	0.08 (0.00, 0.16)	0.050
Wave 15 (2020)	5.36 (5.28, 5.43)	5.36 (5.32, 5.40)	−0.01 (−0.09, 0.08)	0.875
LSM differences between waves*	−0.12 (−0.18, −0.05)	−0.03 (−0.06, 0.00)	−0.09 (−0.16, −0.01)	0.020
*P* for differences between waves*	<0.001	0.092	0.020	/
**During pandemic vs. Before pandemic**				
Differences in LSM differences between two periods*	−0.04 (−0.15, 0.07)	0.04 (−0.03, 0.10)	−0.07 (−0.20, 0.05)	0.254
*P* for differences in LSM differences between two periods*	0.499	0.253	0.254	/

*Differences were calculated by the linear mixed model, after adjusting for age, sex, race, education, cohabitation status, current smoking, current drinking, physical active, depressive symptoms, status of hypertension, diabetes, stroke, cancer, and chronic lung diseases.

### 3.3. Sensitivity analyses

Subgroup analyses were conducted to explore potential modified effects. We observed that the difference in global cognitive scores between people with and without heart diseases during the pandemic period compared with that during the pre-pandemic period was −0.59 (95% CI: −1.01 to −0.17, *P* = 0.006) among female participants, significantly larger than male participants (0.03, 95% CI: −0.42 to 0.49, *P* = 0.893), and the *P*-value for interaction was 0.047. We also found that the pandemic-related difference in global cognitive scores was −0.58 (95% CI: −0.96 to −0.19, *P* = 0.003) among physical active participants, significantly larger than those physical inactive participants (0.13, 95% CI: −0.39 to 0.65, *P* = 0.616), with a *P*-value for the interaction of 0.030. Neither other covariate nor COVID-19 infection was observed to play a modified role (Supplementary Figure 2).

## 4. Discussion

Leveraging longitudinal data from a nationally representative aging cohort in the United States, we observed that older adults with heart diseases exhibited a greater cognitive decline compared with those without heart diseases during the COVID-19 pandemic, while no significant difference in the change of cognitive function was detected during the pre-pandemic period. After accounting for the existing cognitive difference during the pre-pandemic period, we demonstrated that the magnitude of cognitive difference between people with and without heart diseases was significantly enlarged during the pandemic period.

To our current knowledge, this is one of the largest studies to demonstrate the deterioration in cognitive function during the pandemic among the general older population and, more importantly, the first one to identify an enlarged gap in cognitive function related to the pandemic between people with and without heart diseases. A few studies have suggested that older adults experienced a cognitive decline during the pandemic, although these studies were limited in small sample sizes, convenience samples, or lacking objective cognitive assessments measured before and during the pandemic. French PA-COVID study observed an accelerated cognitive decline during the pandemic, compared with 15 years of cognitive trajectory preceding the pandemic among 263 older adults (15). A Japanese survey of 955 older people reported that social isolation was associated with self-reported cognitive impairment during the pandemic (16), while an online survey of 640 Belgium older adults found only those with depressive symptoms exhibited self-perceived cognitive decline during the pandemic, and this study sample mainly focused on individuals with high socioeconomic status (17). Data collected by online surveys inclined to rule out disadvantaged people who do not possess Internet access (29). Our study observed pandemic-related cognitive decline in the both heart disease group and the non-heart disease group, together with these previous findings, emphasizing that increased attention should be paid to cognitive decline among older adults during the pandemic. Moreover, our results showed that the cognitive function gap between people with and without heart diseases significantly grow further during the pandemic. Identifying this vulnerable group is of pivotal importance to provide targeted cognitive monitoring and training as the pandemic progressed.

Our subgroup analyses identified that sex might play a potential modified role in the pandemic-related cognitive difference between people with and without heart diseases, and a larger cognitive difference was presented among female participants. Similarly, previous findings have shown that women were especially susceptible to mental disorders during the pandemic (7, 30, 31). This evidence indicated the sex disparities related to the COVID-19 pandemic and underscored the importance to support vulnerable women. In addition, we also observed that a significantly smaller cognitive difference was exhibited among physical inactive participants, probably because these participants had a much lower cognitive function at the baseline due to their poor health status, with a global cognitive score of 15.04 points at wave 14 in the physical inactive group while 16.21 points in the physical active group (data not shown). Therefore, physical inactive participants were likely to have less room to decline on the cognitive test (32). A more sophisticated cognitive assessment in the future study might help clarify this question.

The atherosclerotic process and induced hypoxic–ischemic brain injury have been well documented to link heart diseases and cognitive decline (33). In addition, the shared vascular factors could also contribute to cognitive decline through multiple biological pathways, such as oxidative stress and inflammation responses (34, 35). It is plausible that the enlarged cognitive gaps between people with and without heart diseases during the pandemic might be attributed to COVID-19 infection (36). The presence of heart disease was associated with a more severe course and higher mortality of COVID-19. The infection could in turn lead to cardiac complications such as myocarditis, arrhythmia, and heart failure, as well as lasting cognitive deficits (37, 38), whereas the proportion of patients with COVID-19 in the present study was too small (2.6%, data not shown) to detect the cognitive decline directly due to COVID-19 infection. Therefore, the deleterious impact of the pandemic on health service access and lifestyle changes was more likely to account for our findings. The nationally representative data from the UK showed that the incident use of cardiovascular disease medicines has drastically decreased compared with the pre-pandemic level, and such missed treatment was estimated to result in more than 13,000 additional cardiovascular disease events (39). In addition, several studies have indicated a decrease in hospitalization rate in patients with heart failure during the pandemic compared with 2019, and the admitted patients exhibited significantly more severe symptoms and higher mortality (40, 41). The diminished access to healthcare might be partly because most outpatient visits and cardiac activities have been deferred or canceled to guarantee the capacity for the care of patients with COVID-19 (5, 42) and partly because people avoided seeking medical care for fear of getting infected (43, 44). The deterioration of prognosis compounded by stress and anxiety during the pandemic made older adults with heart diseases more vulnerable to cognitive decline. Moreover, the social isolation caused by quarantine has exacerbated cardiovascular risk factors such as physical inactivity, obesity, and unhealthy food habits (45). For example, Beydoun et al. found that among the HRS participants, the onset of the COVID-19 pandemic was associated with increased BMI, elevated numbers of cardiometabolic risk factors, and chronic morbidities (10). Taken together, all these repercussions of the COVID-19 pandemic on cardiovascular health could further exacerbate cognitive decline among older adults with heart diseases.

The present study draws strength from the nationally representative longitudinal cohort to provide a comprehensive picture of the COVID-19 pandemic as a determinant of aging issues regarding cognitive decline. By employing objective assessments of cognitive function measured during a similar period preceding the pandemic as control, we were able to unpack and compare the pandemic-related cognitive decline between participants with and without heart diseases.

## 5. Limitations

Nevertheless, our findings should be interpreted with caution given the following limitations. First, the ascertainment of heart diseases was based on self-reported doctor diagnoses, which might lead to a misclassification of heart disease cases and bias our findings to a null. In addition, due to the relatively low response rates of questions on specific heart disease types in the HRS, we were not able to further explore whether the observed associations differed by heart disease types. Second, the cognitive assessment was less elaborate given the large-scale population-based setting. Third, although multiple important covariates have been adjusted, other unmeasured and unavailable determinants such as genetic susceptibility and dietary intake were likely to confound our results. Fourth, 7,842 participants from the HRS wave 14 survey were excluded due to incomplete cognitive data or pre-existed dementia, non-response analyses showed significant differences in characteristics between individuals included and excluded, selection bias could not be ruled out, and the generalizability of our findings might be compromised.

## 6. Future directions

Further investigations with more comprehensive measurements on the diagnosis of heart diseases might yield more accurate estimations and provide more information. In addition, using a more sophisticated neuropsychological assessment might provide insights into other cognitive domains and have a higher capacity to detect more subtle cognitive decline. Furthermore, future studies conducted in non-U.S. populations, with a longer follow-up time during the pandemic, are warranted to verify our findings. Moreover, future policy and guidance should be in place for the immediate provision of cognitive monitoring and interventions for the vulnerable population with heart diseases to mitigate the adverse impact of the pandemic.

## 7. Conclusion

In conclusion, this study illustrated the deteriorated cognitive status among older adults and an enlarged gap in cognitive function between people with and without heart diseases related to the COVID-19 pandemic. The findings underscore the necessity to provide immediate cognitive monitoring and interventions for the population with heart diseases.

## Data availability statement

Publicly available datasets were analyzed in this study. This data can be found here: https://hrsonline.isr.umich.edu/.

## Ethics statement

The Health and Retirement Study was approved by the Institutional Reviewing Board at the University of Michigan and the National Institute on Aging (HUM00061128). The participants provided their written informed consent to participate in this study.

## Author contributions

FZ and WX conceived and designed the study, obtained the funding, had full access to all of the data in the study, and took responsibility for the integrity of the data and the accuracy of the data analysis. RH, CL, DG, and WX performed the statistical analysis. RH, CL, WX, and FZ drafted and revised the manuscript. All authors contributed to the data interpretation and final approval of the manuscript.
