# Supplementary material for: Cognitive decline among older adults with heart diseases before and during the COVID-19 pandemic: A longitudinal cohort study
Source: Front Cardiovasc Med. 2023 Jan 26;9:1077800. doi: 10.3389/fcvm.2022.1077800 (PMC9908755; doi:10.3389/fcvm.2022.1077800)

Supplementary Material

Supplementary Figure 1. Flowchart of participants selection.

Supplementary Table 1. Comparison of baseline characteristics between participants included (n=9304) and excluded (n=7842).

Supplementary Figure 2. Subgroup analyses on the differences in global cognitive scores between people with and without heart diseases during the pandemic period compared with those during the pre-pandemic period.

Supplementary Figure 1. Flowchart of participants selection

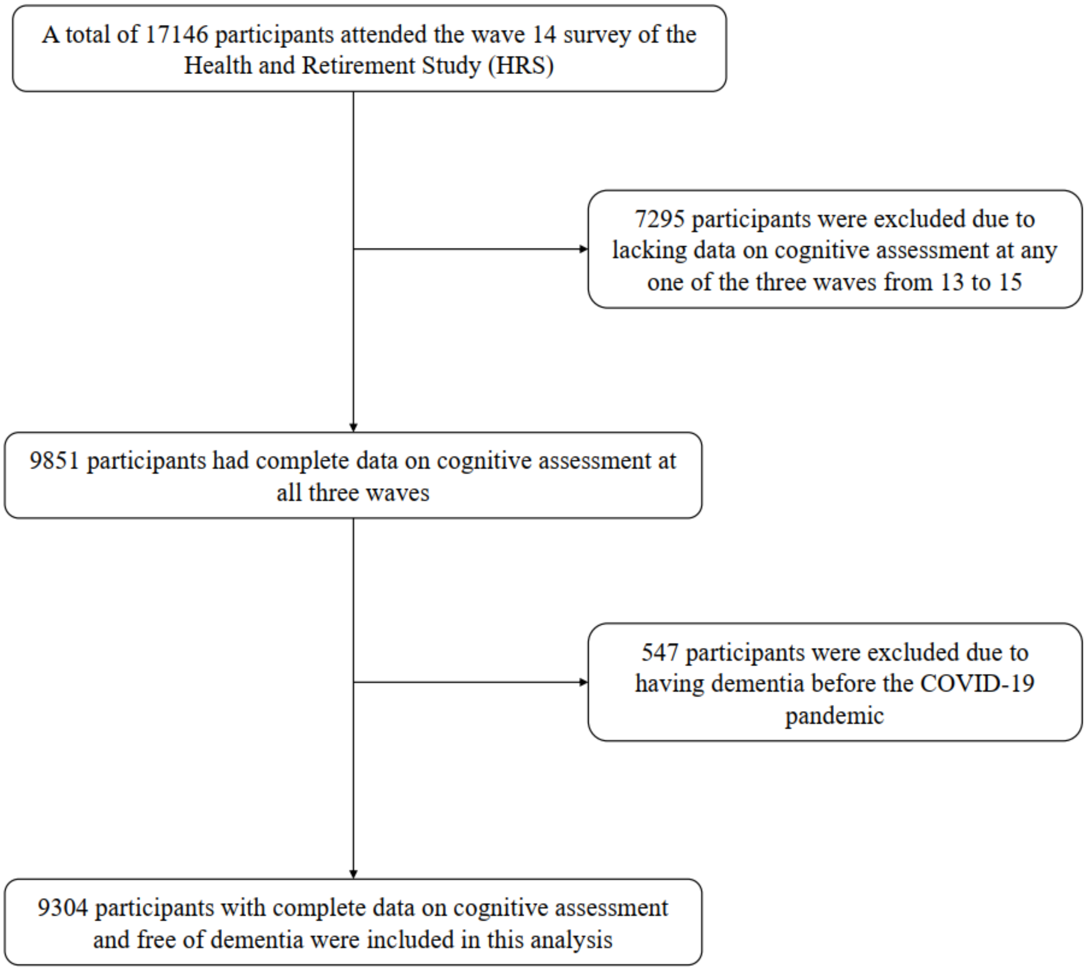

Supplementary Table 1. Comparison of baseline characteristics between participants included (n=9304) and excluded (n=7842)

Characteristics	Included participants (n=9304)	Exclude participants (n=7842)	*P* *
Heart diseases (%)	2119 (22.8)	2124 (27.1)	<0.001
Age (years)	65.8 ± 10.8	68.4 ± 11.9	<0.001
Female (%)	5655 (60.8)	4461 (56.9)	<0.001
White (%)	5825 (62.9)	5470 (70.0)	0.001
High educational level (%)	7506 (80.7)	6310 (80.5)	0.728
Living alone (%)	3521 (37.8)	3163 (40.3)	<0.001
Current smoking (%)	1259 (13.5)	989 (12.6)	0.079
Current drinking (%)	3612 (39.0)	3053 (39.1)	0.863
Physical active (%)	6562 (70.5)	5097 (65.0)	<0.001
Depressive symptoms (%)	1297 (13.9)	1190 (15.2)	0.024
Chronic diseases status			
Hypertension (%)	6268 (67.4)	5342 (68.1)	0.302
Diabetes (%)	2943 (31.6)	2362 (30.1)	0.034
Stroke (%)	701 (7.5)	887 (11.3%)	<0.001
Cancer (%)	1292 (13.9)	1367 (17.5)	<0.001
Chronic lung diseases (%)	971 (10.4)	989 (12.7)	<0.001

Values were presented as mean ± SD, or n (%).

* The differences between included participants and excluded participants were tested using the t-test or chi-square test.

Supplementary Figure 2. Subgroup analyses on the differences in global cognitive scores between people with and without heart diseases during the pandemic period compared with those during the pre-pandemic period.

* The participant self, or his relatives or friends had been diagnosed with COVID-19.

† Calculated by linear mixed model, after adjusting for age, sex, race, education, cohabitation status, current smoking, current drinking, physical active, depressive symptoms, status of hypertension, diabetes, stroke, cancer and chronic lung diseases, except where the adjusting variable itself being tested.

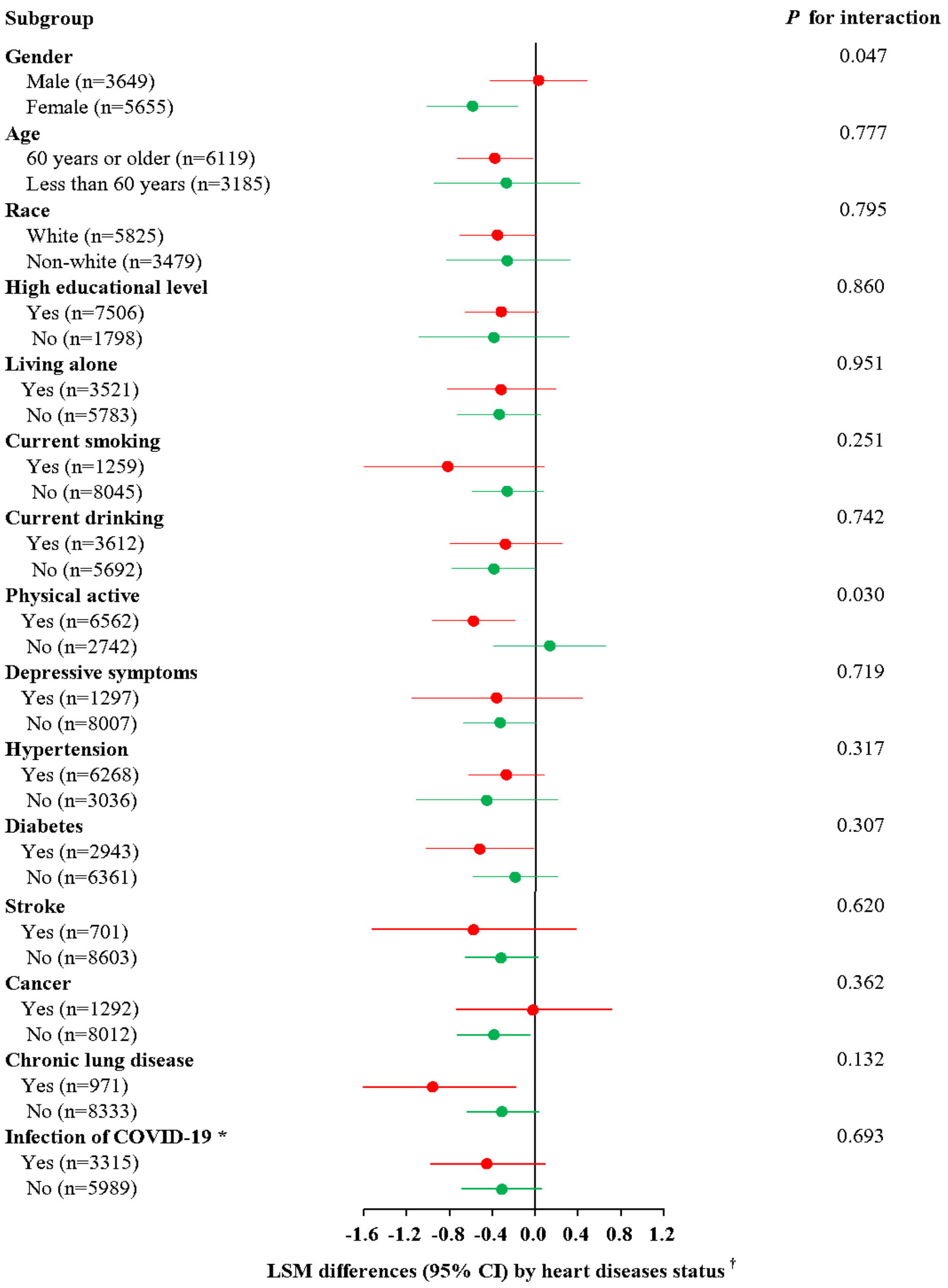

Supplement: Supplementary file 1 [file Data_Sheet_1.pdf]
